# Interictal abnormal fMRI activation of visual areas during a motor task cued by visual stimuli in migraine

**DOI:** 10.1590/S1679-45082017AO3719

**Published:** 2017

**Authors:** Adriana Bastos Conforto, Khallil Taverna Chaim, Mario Fernando Prieto Peres, André Leite Gonçalves, Inara Laurindo Siqueira, Maria Angela Maramaldo Barreiros, Edson Amaro

**Affiliations:** 1 Hospital Israelita Albert Einstein, São Paulo, SP, Brazil.

**Keywords:** Visual cortex, Migraine disorders, Brain mapping, Cerebral cortex, Light

## Abstract

**Objective:**

To assess changes in blood-oxygen-level-dependent activity after light deprivation compared to regular light exposure in subjects with migraine in the interictal state and in controls.

**Methods:**

Ten subjects with migraine and ten controls participated in two sessions of functional magnetic resonance imaging. In each session, they performed a finger-tapping task with the right hand, cued by visual stimuli. They were scanned before and after 30 minutes of light deprivation or light exposure. In subjects with migraine, functional magnetic resonance imaging was performed interictally. Analysis of variance was made with the factors time (before or after), session (light deprivation or exposure), and group (migraine or control).

**Results:**

There were significant “group” effects in a cluster in the bilateral cuneus encompassing the superior border of the calcarine sulcus and extrastriate cortex. There were no significant effects of “time”, “session”, or interactions between these factors.

**Conclusion:**

The main result of this study is consistent with aberrant interictal processing of visual information in migraine. Light deprivation did not modulate functional magnetic resonance imaging activity in subjects with or without migraine.

## RESUMO

**Objetivo:**

Avaliar mudanças na atividade cerebral por meio de ressonância magnética funcional após privação luminosa comparada à exposição à luz, em indivíduos com enxaqueca no estado interictal e em controles.

**Métodos:**

Dez indivíduos com enxaqueca e dez controles participaram de duas sessões de ressonância magnética funcional. Em cada sessão, realizaram uma tarefa motora com a mão direita guiada por estímulos visuais. Foram colhidas imagens antes e após 30 minutos de privação luminosa ou exposição à luz. Em indivíduos com enxaqueca, a ressonância funcional foi realizada no período interictal. Foi feita a análise de variância com fatores tempo (antes ou depois), sessão (privação ou exposição à luz) e grupo (enxaqueca ou controle).

**Resultados:**

Houve efeitos significativos de “grupo” em uma área no cúneo bilateral, incluindo a borda superior do sulco calcarino e o córtex extraestriado. Não houve efeitos significativos de “tempo”, “sessão” ou interações entre estes fatores.

**Conclusão:**

O principal resultado deste estudo sugere um processamento interictal anormal das informações visuais em indivíduos com enxaqueca. A privação luminosa não modulou a atividade na ressonância magnética funcional em indivíduos com ou sem enxaqueca.

## INTRODUCTION

Migraine is a common neurologic condition, with a mean prevalence of 12% in adults.^1^ A widespread concept is that migraine is a disorder of the brain characterized by paroxysmal aberrant sensory processing.^2^ Abnormal visual processing in particular has been observed in migraineurs in behavioral, neurophysiologic, and imaging studies.

The interest on the relation between migraine pathogenesis and visual function was boosted by similarities found between migraine attacks and the cortical spreading depression, phenomenon. Initially described in animals, cortical spreading depression starts in visual areas of the brain. A wave of neuronal depression spreads from the occipital lobe to anterior regions and is paralleled by an initial brief decrease, followed by an increase, and finally, a long-lasting decrease in cerebral blood flow. Similar changes in blood flood were reported in the visual cortex during the attack in patients with migraine with or without aura.^3,4^ Importantly, there is evidence that functional changes in areas related to visual processing are not only temporarily associated to the visual aura during a migraine attack, but may also be observed interictally.

Between attacks, increased amplitudes and lack of habituation of visual evoked potential responses can be observed in subjects with migraine, with or without aura.^5^ In migraine with aura, interictal transcranial magnetic stimulation (TMS) studies indicate increased visual cortical excitability evaluated by phosphene thresholds after stimulation of V1.^6^ Decreased phosphene thresholds obtained after stimulation of V5,^7^ as well as responsiveness to transcranial direct current stimulation of V5,^8^ suggest hyperexcitability of this non-primary visual area in migraine with or without aura. Another evidence in favor of abnormal secondary visual processing is the observation that motion perception is impaired in migraineurs compared to controls.^9^

Furthermore, aberrant responsiveness to sensory stimuli has been documented by decreased preactivation and lack of habituation to afferent input,^5^ as well as by discomfort elicited by visual stimuli, such as stripes or checkerboard patterns^10^ in subjects with migraine. Increased sensitivity to light in migraineurs and improvement of migraine attacks provided by resting in a dark room are also widely known.^11^

Light deprivation (LD) can modulate the functional magnetic resonance imaging (fMRI) hemodynamic response in the primary visual cortex when compared to light exposure (LE).^12^ In subjects with migraine, interictal or ictal abnormalities can be captured in primary and secondary visual areas when visual stimuli are presented in fMRI paradigms.^13^ In addition, LD may increase excitability of the primary motor cortex to TMS in healthy subjects.^14^ Whether modulation of the fMRI hemodynamic response by LD or LE is also abnormal, remains to be determined.

## OBJECTIVE

Our goal in this hypothesis-generating study was to preliminarily compare differences between hemodynamic responses evaluated by functional magnetic resonance imaging, in patients with episodic migraine in the interictal state and in controls, before and after light deprivation or light exposure, during performance of an one-hand motor task guided by visual instructions. We hypothesized that effects of light deprivation on the blood-oxygenation-level dependent signal would differ in patients with migraine and in controls.

## METHODS

### Subjects

Subjects with or without migraine underwent two fMRI sessions on separate days. In one session, fMRI was performed before and after LD, while in the other session, before and after LE. The protocol was approved by our institution’s Ethics Committee protocol number 06/473, CAAE: 0095.0.028.000-06 and complied to the ethical standards described in the Declaration of Helsinki. All subjects provided Informed Consent to participate.

Subjects who wished to participate after receiving information about the protocol from announcements published in local media, from their physicians, or from the researchers, were evaluated by a neurologist. The inclusion criteria for subjects were diagnosis of migraine (with aura, without aura, or chronic) made by a neurologist, according to the International Headache Society (IHS) criteria,^15^ and at least one migraine attack in the month before the experiments. Exclusion criteria for all subjects were: left-handedness according to the Oldfield inventory;^16^ contraindications to MRI; psychiatric conditions other than anxiety or depression; neurological conditions; use of prophylactic migraine drugs in the previous 4 weeks (beta-blockers, calcium channel blockers, antidepressants, or antiepileptic drugs); abnormal brain magnetic resonance imaging: vascular anomalies associated with changes in brain perfusion, compression of venous structures, areas of recent ischemia. Potential control subjects were excluded if they had a history of any headache during their lifetime that fulfilled criteria for a migraine attack according to IHS criteria, any primary headache other than episodic tension-type headache, or any headache during the month before the experiments. All patients with migraine were women; therefore, the male sex was an exclusion criterion for controls in order to avoid differences in sex composition between the groups.

### Functional magnetic resonance imaging

#### Image acquisition

**Figure 1:**
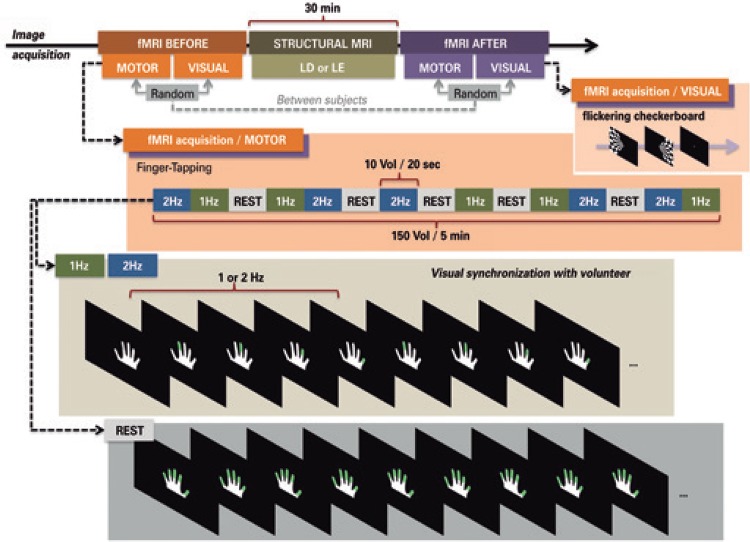
Experimental paradigm. In the motor paradigm cued by visual stimuli, right hand finger tapping at 1 and 2Hz was alternated with rest every 20s in a blocked design. Total run time was 300s, during which five epochs of each condition were sampled. Subjects were instructed to remain with eyes open, fixating the center of a screen, and to keep the right hand at rest with slight wrist flexion. Directions to oppose each finger to the thumb following a specific order were provided visually via well-fitted light-proof goggles. A figure of a hand was presented and subjects had to move fingers colored in green. During the rest condition, all fingers were highlighted. In the visual paradigm, a flickering checkerboard (frequency, 8Hz) was presented to the left hemi-field for 20s, and to the right hemi-field for 20s. A central fixation point was then presented for 20s. The order of the visuomotor paradigm and of the visual paradigm was randomized across subjects

Functional magnetic resonance imaging was performed on a 3T MR scanner (Tim Trio, Siemens, Germany), equipped with a 12-channel head coil. Whole-brain sagittal structural tidimensional T1 MPRAGE (TR=25s, TE=3.45ms, FA 7^o^, 1mm isotropic voxels) and axial FLAIR (TR=9,000ms, TE=81ms, IR=2,500ms, FA 150^o^, matrix 256 x 239, FOV=220mm, slice thickness 6mm) were performed in all subjects. Two sets of 150 functional images (EPI GRE T2* – BOLD; TR=2,000ms, TE=30ms, FA 90^o^, 3.3mm isotropic voxels) were acquired in each subject before and after 30 minutes of LD or LE (Figure 1).

fMRI: functional magnetic resonance imaging; LD: light deprivation; LE: light exposure.

Light-proof goggles were mounted on the head before starting the experiment and were positioned on the eyes during LD. During LE, subjects were only exposed to standard room lighting conditions (530lux). Subjects listened to standard songs during the 30 minutes of LD or LE, and were instructed to remain awake during the experiment. The order of LD and LE sessions was randomized and counterbalanced across subjects.

Visual stimuli were presented binocularly via goggles (NNL, Norway) with a dedicated algorithm (E-prime, Psylab, USA), synchronizing image acquisition with stimulus presentation. In order to minimize head movement, headbands were used. All subjects trained the motor task for up to 20 minutes before entering the scanner.

#### Paradigm

In the motor paradigm guided by visual stimuli, right hand finger tapping at 1 and 2Hz was alternated with rest at every 20s in a blocked design (Figure 1). Total run time was 300s, during which five epochs of each condition were sampled. Subjects were instructed to remain with eyes open, fixating the center of a screen, and to keep the right hand at rest with slight wrist flexion. Directions to oppose each finger to the thumb, following a specific order, were provided visually via well-fitted light-proof goggles. A figure of a hand was presented in white (encompassing 10.6**°**/7.2**°** of the visual field – corresponding to 90/61% of the foveal field; 100% contrast with the background) and subjects had to move fingers colored in green (Figure 1). On average, each finger was projected in 2.7**°**/1.3**°** of the visual field – corresponding to 23/11% of the foveal field; 53% contrast against the background in the foveal region.

The finger that should touch the thumb was highlighted at frequencies of 1 and 2Hz. During the rest condition, all fingers were highlighted, alternating the right and left hand representations (right/left hand visual field difference was 11.9/6.5; 94% contrast against the background in the foveal region). We will show results obtained during finger-tapping at 2Hz, compared to rest; 1Hz, compared to rest.

In addition, blocks of visual stimuli were presented before and after LD. Visual stimuli consisted of a checkerboard pattern at a frequency of 8Hz, presented to the left hemifield for 20s, to the right hemifield for 20s, and to a central fixation point for 20s. These conditions were randomized across subjects. The order of presentation of visual and visuomotor stimuli was also randomized.

#### Image analysis

Image processing and data analysis were performed using the FMRIB software library package FSL (Analysis group, FMRIB, Oxford, UK, http://www.fmrib.ox.ac.uk/fsl/). Standard pre-processing was done with Motion Correction FMRIB’s Linear Image Registration Tool (MCFLIRT) – slice time correction/motion correction, Brain Extraction Tool – brain extraction, time-series pre-whitening, registration and spatial normalization to the Montreal Neurological Institute (MNI) high-resolution 152-T1 2mm template. Images were resampled into this space with 2-mm isotropic voxels. A smoothed filter was applied with a Gaussian kernel of full-width at half-maximum (FWHM=5mm) to minimize noise and residual differences in gyral anatomy. A high-pass filter (40s) was used to remove high frequency noise. Statistical inferences were based on the theory of random Gaussian fields, and changes relative to the paradigms were modeled by convolution of single trial epochs with the canonical Hemodynamic Response Function to approximate the activation patterns with the FMRIB’s improved linear model (FILM)*.* Using multiple regression analysis, statistical maps representing the association between the observed time series (*e.g*., BOLD signal) and a linear combination of regressors for each subject were constructed. Results of the motor paradigm will be presented in this manuscript. Results of the visual paradigm will be present elsewhere.

Group analysis was performed using the higher level FEAT analysis tool to yield statistical parameter maps (SPM) in which all subsequent analyses were performed. Analysis of Variance (ANOVA) analysis with factors time (before or after), session (LD or LE), and group (patients with migraine or controls) was implemented using the Design Matrix feature with the contrasts finger-tapping and rest, and SPM were thresholded on a voxel-wise basis at *Z=*2.3, and a (corrected) cluster significance threshold of p=0.05. The maximum change in BOLD signal was collected in the “local maxima” of areas in which significant effects or interactions were identified in the ANOVA, for post-hoc analysis. MRI3DX version 7.63 was used for visual analysis and tridimensional rendering.

Because this was a hypothesis-generating study, the sample size was not formally determined. Results of this study may be used to plan larger studies in the future.

## RESULTS

### Subjects

Twenty women participated in the study: ten subjects with migraine (mean age ±standard deviation, 34.8±7.7 years) and ten controls (33.9±12.8 years). All subjects were right-handed. Oldfield^16^ Inventory scores for patients ranged from 58 to 100, and for controls, from 50 to 100.

Six patients had migraine without aura, and four, with aura. Migraine history averaged (±standard deviation) 20.6±9.4 years, and the mean number of days with pain was 10.4±9.4 per month. Median Migraine Disability Assessment Score (MIDAS) was 33.5 (1-80). MIDAS scores higher than 20 indicate severe disability. The average intervals between the last migraine attack and fMRI sessions were 6.1±5.1 days (LE session) and 8.6±7.4 days (LD session).

### Functional magnetic resonance imaging

**Figure 2:**
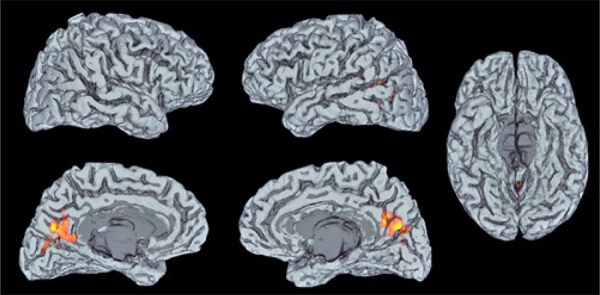
Brain regions with significant differences for the contrast (2Hz finger-tapping cued by visual stimuli > rest) in subjects with and without migraine: the cluster in the bilateral cuneus encompasses the superior border of the calcarine sulcus (V1, BA 17) and extrastriate cortex (V2, BA 18). Statistical parameter maps were thresholded on a voxel-wise basis at Z=2.3 and a (corrected) cluster significance threshold of p=0.05

**Figure 3:**
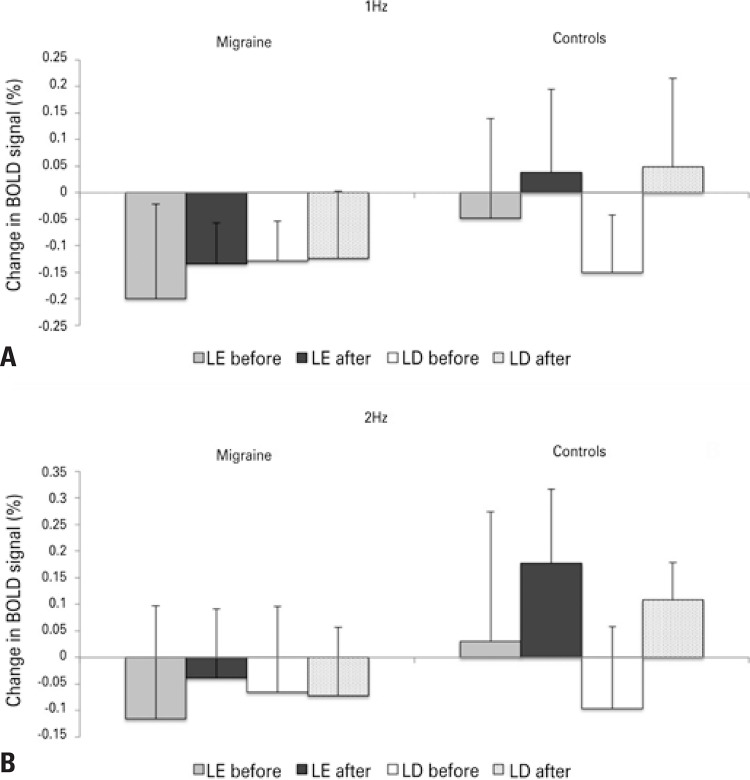
Variation in BOLD signal (%, contrast between finger-tapping, and rest) in patients with migraine (left) and controls (right) before and after light exposure and light deprivation, during finger tapping at 1Hz (A) and 2Hz (B). Mean percentage is shown by each bar. Error bar = standard deviation of the mean. Measurements were made in the local maxima of the region encompassing the superior border of the calcarine sulcus (V1, BA 17) and extrastriate cortex (V2, BA 18). x=0 y=-90 z=24

LD: light deprivation; LE: to light exposure.

Table 1 and figure 2 show significant ANOVA_RM_ effects or interactions at 2Hz and 1Hz. There were significant “group” effects in a cluster in the bilateral cuneus encompassing the superior border of the calcarine sulcus (V1, BA 17) and extra-striate cortex (V2, BA 18). There were no significant effects of “time”, “session”, or interactions between these factors. Figure 3 shows the results of post-hoc analysis.

**Table 1:** Summary of the significant effects in functional magnetic resonance imaging activation for the motor paradigm, at finger-tapping frequencies of 1Hz and 2Hz. Areas, Z scores, volumes (in mm3), and coordinates of the local maximal of activation are shown

Condition	Area (side)	Z score	mm^3^	x	y	z
Group effect 1Hz	Lingual gyrus (L)	4.17	5372	-14	-92	-6
	Superior temporal gyrus (R)	3.92	4210	52	-12	-8
	Paracentral lobule (L)	3.76	1770	-14	-24	58
	Inferior occipital gyrus (R)	3.11	729	42	-72	-8

Group effect 2Hz	Left cuneus	4.24	4006	-1	-90	24

L: left; R: right.

## DISCUSSION

The main finding of this study was the significant difference between migraineurs in the interictal state and controls in fMRI activation in primary and extrastriate visual cortex during a finger-tapping task cued by visual stimuli, compared to rest. This finding is consistent with evidence of interictal aberrant processing of visual information in migraineurs. Our hypothesis of differential modulation of the BOLD signal in the visual cortex by LD was not confirmed. However, due to the relatively small sample size we cannot completely exclude this assumption.

Most interictal functional neuroimaging studies in migraine evaluated responses to painful stimuli.^17^ Our paradigm did not involve presentation of painful stimuli and our results showed, for the first time, interictal abnormal activity in striate and extrastriate areas in subjects with migraine during a motor task cued by visual stimuli. Enhanced activity in another area, the peristriate visual cortex, has been reported in subjects with migraine with aura in an fMRI block paradigm in which incongruent lines were visually presented.^18^ Also, in patients with fixed-side migraine aura, interictal fMRI showed enhanced BOLD responses in the symptomatic hemispheres during visual stimulation.^19^

Abnormal interictal activity in brain areas may be specific to paradigms employed to address particular aspects of visual processing. For instance, no interictal H_2_^15^O PET changes were observed in patients with migraine in the interictal state, compared to controls, in response to photostimulation, while increased activation was described during attacks.^4^ Photostimulation is a robust paradigm to elicit changes of the hemodynamic response in the visual cortex, but may not capture differences in operation of specific networks between migraineurs and controls.

We found no significant changes in fMRI activation in sensorimotor areas. Enhanced activation of the contralateral sensorimotor cortex in subjects with migraine without aura, compared to controls, has been reported when a hand motor paradigm is used in a block design.^20^ The specificities of the paradigm may explain this discrepancy: Rocca et al.^20^ chose a motor task consisting of 1-Hz flexion and extension of the last four digits of the right hand, alternated with rest. In contrast with the visual presentation of the stimulus in our paradigm, auditory cueing was applied with a metronome to pace movement frequency, and fMRI was performed in a 1.5T scanner. Interictal fMRI connectivity studies point to an interictal network disorder in migraine, rather than to dysfunction restricted to primary and secondary sensory areas.^2,17,21-28^ Auditory or visual cues may engage different networks, and hence have distinct abilities to show anomalous activation in visual or sensorimotor areas, in a condition as complex as migraine.

“Increased” or “decreased” activation of visual areas may be an oversimplification of subtle mechanisms of deviations from normality in the brains of patients with migraine. In the present study, the changes in activity were different in migraineurs and controls in fMRI measurements, when performed twice within an experimental session. Changes in activation or responsiveness over time may be a more robust endpoint for comparisons between subjects with or without migraine, than evaluation of these parameters at a particular point in time.

An example of different responsiveness over time in subjects with or without migraine is the phenomenon of lack of habituation. For instance, when paired face stimuli are presented to healthy subjects, a decrease in hemodynamic response can be observed, compared to when a single face is shown in an event-related design. Interictal, event-related fMRI with paired visual stimuli revealed a defective pattern of habituation of the hemodynamic response in migraineurs,^28^ in line with the previously described absence of habituation of visual evoked responses reported in subjects with migraine with or without aura, between attacks.^5^ In addition, changes in resting motor thresholds to TMS during a period of rest were reported over time in patients with migraine, whereas no significant changes were observed in control subjects.^27^

Our fMRI results, obtained by using a non-painful motor paradigm cued by visual stimuli, support the concept of interictal abnormal visual function in migraine. It is believed that migraine attacks are generated by changes in the brain, including abnormal cortical excitability.^5,27^ Possibly, abnormal processing in visual or other sensory areas could lead to activation of the “pain matrix” involving the thalamus, insula, anterior cingulate cortex, prefrontal cortex, primary and secondary sensory cortex, and the cerebellum.

Light deprivation, when compared with LE, did not lead to changes in fMRI activation in subjects with or without migraine. A duration of 60 minutes of LD increased fMRI activation of the visual cortex and decreased phosphene thresholds measured with TMS in healthy subjects.^12^ The choice of a 30-minute duration in the present study was based on a report of increased corticomotor excitability to TMS, after 30 minutes of LD, compared to baseline.^14^ However, TMS experiments performed in parallel to the present fMRI study failed to show changes in corticomotor excitability after 30 minutes of LD relative to baseline, when compared to changes after LE relative to baseline, in migraineurs or controls.^28^ Considering that rest influences baseline activity in the brain, the 30-minute period of relative rest during LD or exposure may have exceeded possible effects of light modulation on fMRI activation or on excitability to TMS. Thus, any differences in effects of LD, when compared to LE, may have been obscured.

The main limitation of this study is the sample size. It was not possible to perform subgroup analysis in order to address possible differences in results between patients with migraine with or without aura, or whether there is a correlation with the frequency of attacks, extent of disability from migraine, and abnormal sensory processing. Further studies are necessary to clarify these points and to investigate whether the fMRI results can predict risk of headache chronification or responsiveness to specific treatments.

## CONCLUSION

Interictal abnormal function and subtle structural abnormalities in the visual cortex may reflect plastic processes related to repeated migraine attacks, as well as associated modifications in blood flow or metabolism. Alternatively, increased thickness or aberrant interictal activity in cortical sensory areas may reflect a common mechanism with a likely strong genetic component, leading to migraine and to aberrant sensory processing. Prospective studies are necessary to determine if structural and functional findings are already present before the first migraine attack occurs, or if they develop after repeated attacks throughout the life span.
